# Secure Messaging Through PositiveLinks: Examination of Electronic Communication in a Clinic-Affiliated Smartphone App for Patients Living with HIV

**DOI:** 10.1089/tmj.2018.0261

**Published:** 2020-03-04

**Authors:** Tabor E. Flickinger, Karen Ingersoll, Sabrina Swoger, Marika Grabowski, Rebecca Dillingham

**Affiliations:** ^1^ Department of Medicine, University of Virginia School of Medicine, Charlottesville, Virginia.; ^2^ Department of Psychiatry and Neurobehavioral Sciences, University of Virginia School of Medicine, Charlottesville, Virginia.; ^3^ University of Virginia School of Medicine, Charlottesville, Virginia.

**Keywords:** mobile health, smartphone app, HIV/AIDS, patient–provider communication, PositiveLinks, telemedicine

## Abstract

***Purpose:*** Secure messaging between patients and their health care team can facilitate chronic care management. PositiveLinks^®^ (PL) is a clinic-affiliated smartphone application designed for patients living with HIV that includes a secure messaging feature for patients, PL staff, and clinic providers to communicate. Our aim was to examine the content and function of messaging within PL.

***Methodology:*** We examined messages exchanged through PL from November 2017 through January 2018. Qualitative analysis included categorization of topics as: related to the app, medical care, or social needs. Messaging functions were categorized as information exchange or rapport building.

***Results:*** Of the 1,474 PL messages analyzed, 44% were sent by PL staff, 38% by patients, and 18% by providers, whereas 61% were received by patients, 22% by providers, and 17% by PL staff. Message topics included app-related (57.6%), medical care (34.3%), and social concerns (12.4%). App-related messages addressed technical difficulties, software updates, or coordinating phone payments. Medical messages included medical information, medications, appointments, outreach, and care coordination for physical and mental health. Social messages related to insurance, transportation, housing, food, utilities, disability, finances, and work absences. Message function coding showed that 87.3% of messages contained information exchange and 33.8% contained rapport building. Messages sent by providers were most likely to contain rapport building at 54.8%.

***Conclusion:*** PL messaging was used to handle medical and social needs with potential impact on patients' health and offers an opportunity to strengthen patient–provider relationships through responsiveness and rapport building. Secure messaging through a clinic-affiliated smartphone app could enhance patient-centered care between clinical visits.

## Introduction

The use of electronic patient portals is increasing rapidly over time,^1–3^and emerging evidence indicates potential benefits.^4^ Patients have demonstrated improved clinical outcomes in chronic diseases, including diabetes and hypertension, and increased use of preventive health services.^5^ In addition, patients can experience improved empowerment in disease self-management^6,7^ and satisfaction with care.^8,9^ The use of patient portals is associated with higher medication adherence^9,10^ including for patients with HIV.^11^ HIV patients who use secure messaging have a trend toward higher likelihood of achieving viral suppression.^12^

The portal features most valued by patients are patient–provider messaging and access to test results.^13^ Provider use of secure messaging is associated with increased patient engagement with electronic portals.^14^ Patients who engage in secure messaging with providers have higher trust in providers and higher ratings of patient–provider communication.^15^ Electronic portal use is higher among patients who are young, white, and of higher socioeconomic status.^5^ There is a need to improve access for vulnerable patients and those of lower health literacy.^16,17^

PositiveLinks^®^ (PL) is a unique form of secure patient–provider messaging within a smartphone application designed to enhance linkage and retention in HIV care. PL is similar to patient portals in that patients have access to messaging, appointments, and laboratory data. However, PL differs in that patients receive daily queries related to medication adherence, mood, and stress to encourage self-monitoring, and there is a community message board for secure anonymous communication with other patients living with HIV. Our prior work indicates that PL reaches a vulnerable population with a high prevalence of low literacy, low socioeconomic status, and racial/ethnic minority status, all populations who tend to have low uptake of patient portals.^18^ The pilot phase of PL resulted in sustained improvement in engagement in care and viral suppression in this same population.^19^ The pilot phase included the daily queries, appointment reminders, and the community message board and was entirely patient-facing with no provider involvement. In the subsequent phase, we added the secure messaging feature to facilitate communication between PL patients and their HIV care team.

This report investigates how secure messaging, a recently added feature, was used in the PL mobile health intervention. It examines who was exchanging messages, the content of the messages, and the function the messages served. PL is now included in usual care at the University of Virginia Ryan White HIV clinic. This work was performed as a quality improvement project for the purpose of understanding and optimizing the use of secure messaging to support patient care at the clinic.

## Methods

We examined PL messages from November 2017 through the end of January 2018. All messages sent during this time period were included in the analysis, except for test messages sent by staff to check system function. The initial codebook was developed using an open coding strategy, then applied independently by two coders, and refined until excellent reliability was achieved with a kappa statistic of 0.89. The entire sample was coded so that frequencies of codes could be established.

Coding included sender and recipient roles (patient, PL staff, or provider), message topic, and message function. Topic codes categorized messages as app-related or care-related, which could be either medical or social. Function codes categorized messages by whether they served a purpose of information exchange or rapport building. Topic and function codes were not mutually exclusive, as each message could contain more than one type of content.

Qualitative analysis was performed using Dedoose software with descriptive statistics of code frequencies and code co-occurrence. Conversation threads were also examined to gain an understanding of how issues were addressed in these exchanges.

## Results

Demographic and socioeconomic characteristics of patients using the PL messaging during this time period are presented in *Table 1*. A total of 151 patients were enrolled in PL by the end of the examined time period. Of these, 148 patients logged into the app. The average number of messages per week was 108.2 ± 32.8. For patients, the average number of messages sent and received was 9.3 ± 11 over 3 months or 0.72 ± 0.85 messages per patient per week. For providers, the average number of messages sent and received was 52.2 ± 152.2 over 3 months or 4 ± 11.7 messages per provider per week. Of the 1,474 PL messages analyzed, 44% were sent by PL staff, 38% by patients, and 18% by providers, whereas 61% were received by patients, 22% by providers, and 17% by PL staff.

**Table 1.** Demographics of PositiveLinks^®^ Participants Using the Secure Messaging (*n* = 144)

	NUMBER OF PARTICIPANTS	PERCENTAGE
Age in years (SD)	45.6 (11.7)	
Minimum	20	
Maximum	68	
Mean % of federal poverty level (SD)		96.7 (115.8)
Insurance status		
Private—individual	66	46.8
Private—employer	12	8.5
Medicare part A/B	24	17.0
Medicaid	32	22.7
No insurance	7	5.0
Race		
Black or African American	88	65.7
White (non-Hispanic)	46	34.3
Gender		
Male	90	62.5
Female	50	34.7
Transgender male-to-female	3	2.1
Transgender female-to-male	1	0.7

SD, standard deviation.

**Fig. 1.**
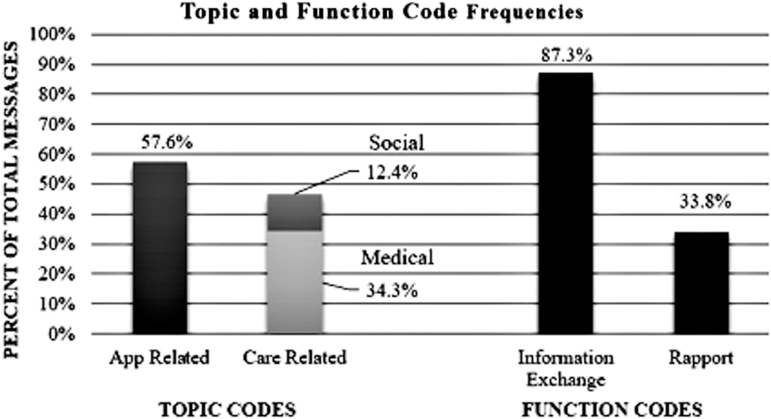
Topic and function code frequencies.

**Fig. 2.**
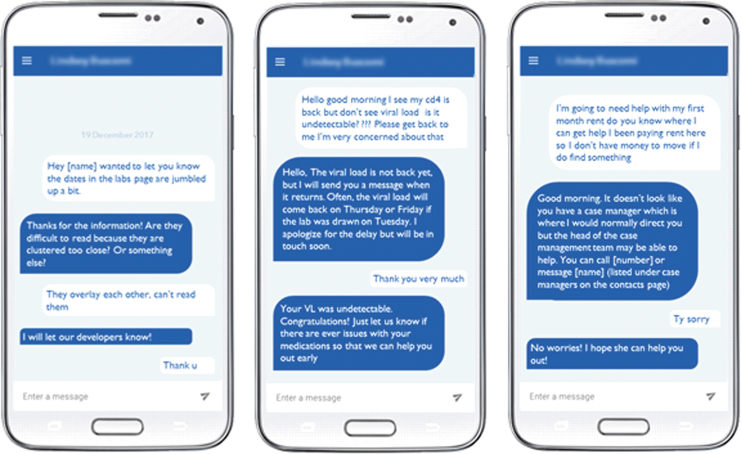
Screenshots of de-identified conversations between patients, PositiveLinks^®^ staff, and clinic providers regarding app-related issues, medical care, and social needs.

Coding showed that 57.6% of messages were app-related, 34.3% related to medical care, and 12.4% related to social concerns. App-related messages included technical difficulties, software updates, or coordinating phone payments. Messages classified as medical related to patient care, medical information, medications, appointments, and outreach, including physical and mental health care coordination. Messages classified as social related to insurance, transportation, housing, food, utilities, disability, finances, and work absences. Regarding function codes, 87.3% of messages contained information exchange and 33.8% contained rapport building. Information exchange messages were utilitarian in nature and served a purpose of coordinating aspects of care or app function. Rapport-building messages included psychosocial components that built relationships, such as addressing emotions, offering thanks or appreciation, or providing more personal content than needed to simply convey information. Topic and function code frequencies are shown in *Figure 1*. Code co-occurrence frequencies by sender and recipient type are shown in *Table 2* with definitions of the code categories. Messages sent by providers were most likely to contain rapport building at 54.8%, followed by patients at 45.5%. *Figure 2* shows typical examples of conversations between patients and PL staff and between patients and providers. The messaging was used not only to support patients in using the app effectively but also to handle medical and social needs with potential impact on patients' health.

**Table 2.** Frequency of Code Occurrences Stratified by Sender

	CODE WITH DEFINITION	PATIENT SENDER (n = 541)	PROVIDER SENDER (n = 248)	PL STAFF SENDER (n = 615)
Recipient	PL staff	44.0% (238)	0.0% (0)	0.7% (4)
	Patient	0.0% (0)	100.0% (248)	99.3% (611)
	Provider	56.0% (303)	0.0% (0)	0.0% (0)
Topic	App related: messages related to managing the PL application or membership. Includes technical difficulties, app payment coordination, meetings/communication with PL staff, and feedback about the app.	40.9% (221)	0.8% (2)	95.3% (586)
	Medical: messages related to patient care in the clinic, including medical information, medications, appointments, and patient outreach. Includes physical and mental health information.	49.0% (265)	78.6% (195)	3.4% (21)
	Social: messages about social aspects of patient health, including insurance, transportation, housing, food, utilities, disability, finances, and work.	15.9% (86)	29.0% (72)	2.6% (16)
Function	Information exchange: messages that are utilitarian in nature and coordinate aspects of care or app function.	77.8% (421)	85.5% (212)	96.3% (592)
	Rapport: messages include a psychosocial component that builds a relationship between users. Used to express emotions and strengthen the connection of the users.	45.5% (136)	54.8% (136)	15.1% (93)

PL, PositiveLinks.

The app-related messages were further classified into subcategories to describe their content more fully. Most app-related messages (74.6%) were logistics messages, which supported the routine use of the app. These included announcements and instructions about app updates and phone payments, for example, “Please use the redemption code below to apply your phone credit.” The PL program provided participants with payment assistance for their phone bills if they met app usage requirements. Each payment code was delivered by secure messaging or in-person by PL staff. Messages about technical difficulties made up 18.0% of app-related messages. Nonspecific feedback about the app was 0.4% of messages (e.g., “This new app is so cool big homie”).

Of the technical difficulties messages, 49% were from patients alerting staff to problems they were having (e.g., “the app is having issues with sending all the alerts 3 times”) and 51% were responses from staff helping resolve the issue (e.g., “our tech guys were wondering if you could try completely uninstalling the app and reinstalling it to see if that resolves the issue of multiple notifications”). The technical difficulties addressed in the messages were duplicate notifications for the medication, mood and stress queries, difficulty with login or getting unintentionally signed out, app slowness, issues with phone service or payment plans, trouble posting on the community message board, and appointments not showing up on the calendar. Problems were addressed as they arose by PL staff who worked with the patient individually if needed or notified the development team about issues affecting multiple users. The development team fixed the issues through software updates, all except for slowness caused by poor Wi-Fi connections, which was out of their control.

## Discussion

PL gives patients and their HIV care team the opportunity to interact through the secure messaging feature of a clinic-affiliated smartphone app. We found that the messaging is used by patients, PL staff, and clinic providers to address both app-related and care-related topics. The messaging appears to serve both an information exchange function and a rapport-building function. On average, providers handled about four PL messages per week, which was not an overwhelming task and allowed them to resolve patient issues in a timely manner between clinic visits.

Although the use of patient portals is increasing and may improve some aspects of patient care, less attention has been paid to the interpersonal aspects of electronic patient–provider communication. A prior study of e-mail messaging between patients and physicians found that most messages were devoted to information exchange, but some were characterized as expressing emotion or responding to emotion and served the purpose of building therapeutic relationships.^20^ A recent analysis of messaging through the Veterans Affairs portal found that most messages included logistical content and were neutral in tone, but some also displayed friendliness, warmth, reassurance, or encouragement, suggesting that this form of electronic communication can build rapport in addition to information exchange.^21^

The majority of app-related messages exchanged through PL were part of the routine functioning of the app, but there were also technical difficulties revealed by the messaging. These difficulties involved features of the app, such as queries, posting, and tracking appointments, or more general issues with phone service or slowness. The messaging gave patients a way to notify PL staff of problems, which were addressed quickly as they arose. The development team provided software updates to improve the app iteratively during implementation and will continue to do so as PL enrollment expands at the clinic.

A limitation of this study is a lack of outcome data relative to the use of secure messaging. The improvements in patient engagement and viral suppression associated with PL were for the pilot phase, before the addition of the secure messaging feature.^19^ Further longitudinal follow-up of current PL participants is planned. Another limitation to consider is generalizability. PL is one app deployed in one clinical setting, which may have unique characteristics. However, PL contributes an example of how a mobile health intervention can be used to facilitate patient–provider communication in a vulnerable patient population, which fills a gap in the literature and seeks to address a need in reducing health disparities. Other developers of mobile health interventions for patient engagement may benefit from our experience in iterative app improvement and consider how technology may be used to help patients overcome barriers to care, including through improvements in patient–provider communication. In addition, our method for examining message content could be applied to other apps with a similar feature to allow comparison of content across platforms.

Secure mobile messaging may help to enhance patient-centered care between clinical visits. PL was designed with patients living with HIV and customized for low literacy, which may make it more accessible to patients with low uptake of health system patient portals. Next steps will include further analysis of patient–provider communication via electronic messaging and examination of potential impact of messaging on provider experiences and attitudes, patient outcomes, and retention in care.
